# *Sca1*-Derived Cells Are a Source of Myocardial Renewal in the Murine Adult Heart

**DOI:** 10.1016/j.stemcr.2013.09.004

**Published:** 2013-10-24

**Authors:** Shizuka Uchida, Piera De Gaspari, Sawa Kostin, Katharina Jenniches, Ayse Kilic, Yasuhiro Izumiya, Ichiro Shiojima, Karsten grosse Kreymborg, Harald Renz, Kenneth Walsh, Thomas Braun

**Affiliations:** 1 Max Planck Institute for Heart and Lung Research, Ludwigstr. 43, 61231 Bad Nauheim, Germany; 2 Institute of Cardiovascular Regeneration, Centre for Molecular Medicine, Goethe-University Frankfurt, Theodor-Stern-Kai 7, 60590 Frankfurt am Main, Germany; 3 Institute of Laboratory Medicine and Pathobiochemistry, Molecular Diagnostics, Philipps University Marburg, Hans-Meerweinstr. 2, 35043 Marburg, Germany; 4 Department of Cardiovascular Medicine, Graduate School of Medical Sciences, Kumamoto University, 860-8556 Kumamoto, Japan; 5 Department of Medicine II, Kansai Medical University, 573-1010 Osaka, Japan; 6 Molecular Cardiology, Whitaker Cardiovascular Institute, Boston University Medical Campus, Boston, MA 02118, USA

## Abstract

Although the mammalian heart is one of the least regenerative organs in the body, recent evidence indicates that the myocardium undergoes a certain degree of renewal to maintain homeostasis during normal aging. However, the cellular origin of cardiomyocyte renewal has remained elusive due to lack of lineage tracing experiments focusing on putative adult cardiac precursor cells. We have generated triple-transgenic mice based on the tet-cre system to identify descendants of cells that have expressed the stem cell marker *Sca1*. We found a significant and lasting contribution of *Sca1*-derived cells to cardiomyocytes during normal aging. Ischemic damage and pressure overload resulted in increased differentiation of *Sca1*-derived cells to the different cell types present in the heart. Our results reveal a source of cells for cardiomyocyte renewal and provide a possible explanation for the limited contribution of *Sca1*-derived cells to myocardial repair under pathological conditions.

## Highlights

• *Sca1*^pos^ cells continuously generate cardiomyocytes during adult life
• Some *Sca1*^pos^ cells are tightly associated with cardiomyocytes
• *Sca1*^pos^-derived cells show limited clonal expansion
• Pressure overload moderately increases the number of *Sca1*-derived cardiomyocytes

## Graphical Abstract

**Figure:**
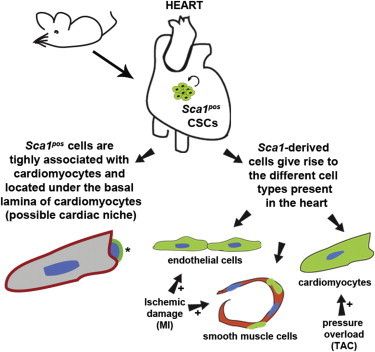

## Teaser

To understand the renewal of cardiomyocytes, Uchida, Braun, and colleagues followed the fates of *Sca1*-positive cells in the heart. By utilizing triple-transgenic mice, they found a significant and lasting contribution of *Sca1*-derived cells to cardiomyocytes during normal aging. Ischemic damage and pressure overload resulted in increased differentiation of *Sca1*-derived cells to the different cell types present in the heart.

## Introduction

Proliferation of mammalian cardiomyocytes decreases after birth, eventually resulting in an arrest of cell division activity (Porrello et al., 2011), although adult cardiomyocytes might enter the cell cycle under certain conditions and undergo endoreduplication (Rumyantsev et al., 1990). Limited proliferation of adult mammalian cardiomyocytes is correlated with a restricted capacity of heart regeneration, whereas fish and certain amphibian species that own proliferating cardiomyocytes can readily regenerate even extensive cardiac injuries (Laflamme and Murry, 2011). Despite the low competence of mammals for heart regeneration, several studies indicate that humans and other mammals create new cardiomyocytes during their lifetime, although no consensus exists about the degree of replacement or the origin of newly formed cardiomyocytes (Bergmann et al., 2009; Kajstura et al., 2010; Hsieh et al., 2007). Recent studies suggest that under normal physiological conditions, murine adult cardiomoycytes are able to give rise to new cardiomoycytes at a rate of ∼1.3%–4% per year (Malliaras et al., 2013), while pathological conditions seem to favor (limited) regeneration of the myocardium both by preexisting cardiomyocytes and by cardiac stem/progenitor cells (CSCs) (Senyo et al., 2013). Numerous attempts have been made to identify CSCs in the rodent heart, resulting in the identification of cell populations that express cell surface markers (e.g., C-KIT and SCA1 proteins), although their long-term contribution to myocardial renewal is unknown due to the absence of cell-tracing studies (Parmacek and Epstein, 2009). In addition, no specific marker has been identified so far that can exclusively label CSCs. While the extent of the contribution of endogenous CSCs to myocardial repair in response to heart injury is unknown, clinical trials based on the use of heart-derived cells have yielded encouraging results (Ptaszek et al., 2012). In this report, we assess the fate of *Sca1*-positive cells in the myocardium using various cell lineage tracing approaches to analyze whether CSCs contribute to the turnover of cardiomyocytes during physiological aging and after myocardial injury.

## Results

### Cardiomyocytes Do Not Express SCA1 in Healthy or Injured Hearts

**Figure 1:**
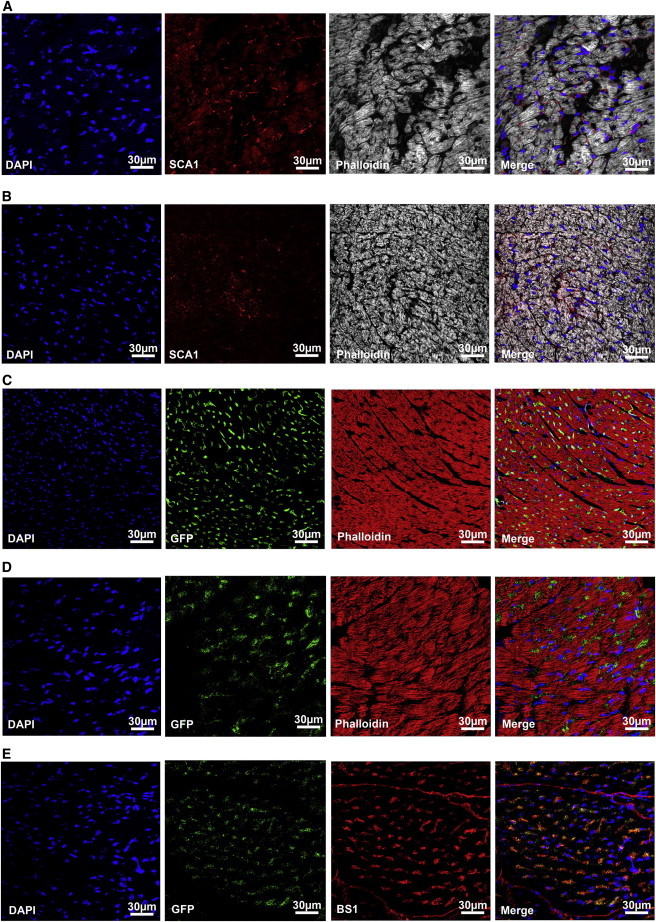
SCA1 Is Not Expressed in Cardiomyocytes (A and B) Cross sections of murine hearts stained with anti-SCA1 antibody (red) and phalloidin (white) to visualize cardiomocytes. (A) Normal heart of a 4-month-old C57BL/6 mouse. (B) Hypertrophied heart of a 4-month-old C57BL/6 mouse, which was challenged with transverse aortic constriction (TAC) for 4 weeks. (C) Cross sections of a normal heart of a 2-month-old *Sca1*-GFP mouse stained with anti-GFP antibody (green) and phalloidin (white) to visualize cardiomocytes. (D) Cross sections of a heart of a 4-month-old *Sca1*-GFP mouse, which was challenged with myocardial infarction (MI) for 4 weeks, stained with anti-GFP antibody (green) to identify *Sca1*-positive cells and phalloidin (red) to identify cardiomyocytes. (E) Cross sections of a heart of a 4-month-old *Sca1*-GFP mouse, which was challenged with MI for 4 weeks, stained with anti-GFP antibody (green) to identify *Sca1*-positive cells and BS-1 (red) to identify endothelial cells. All sections were counterstained with DAPI to visualize nuclei (blue). See also Figure S1.

**Figure 2:**
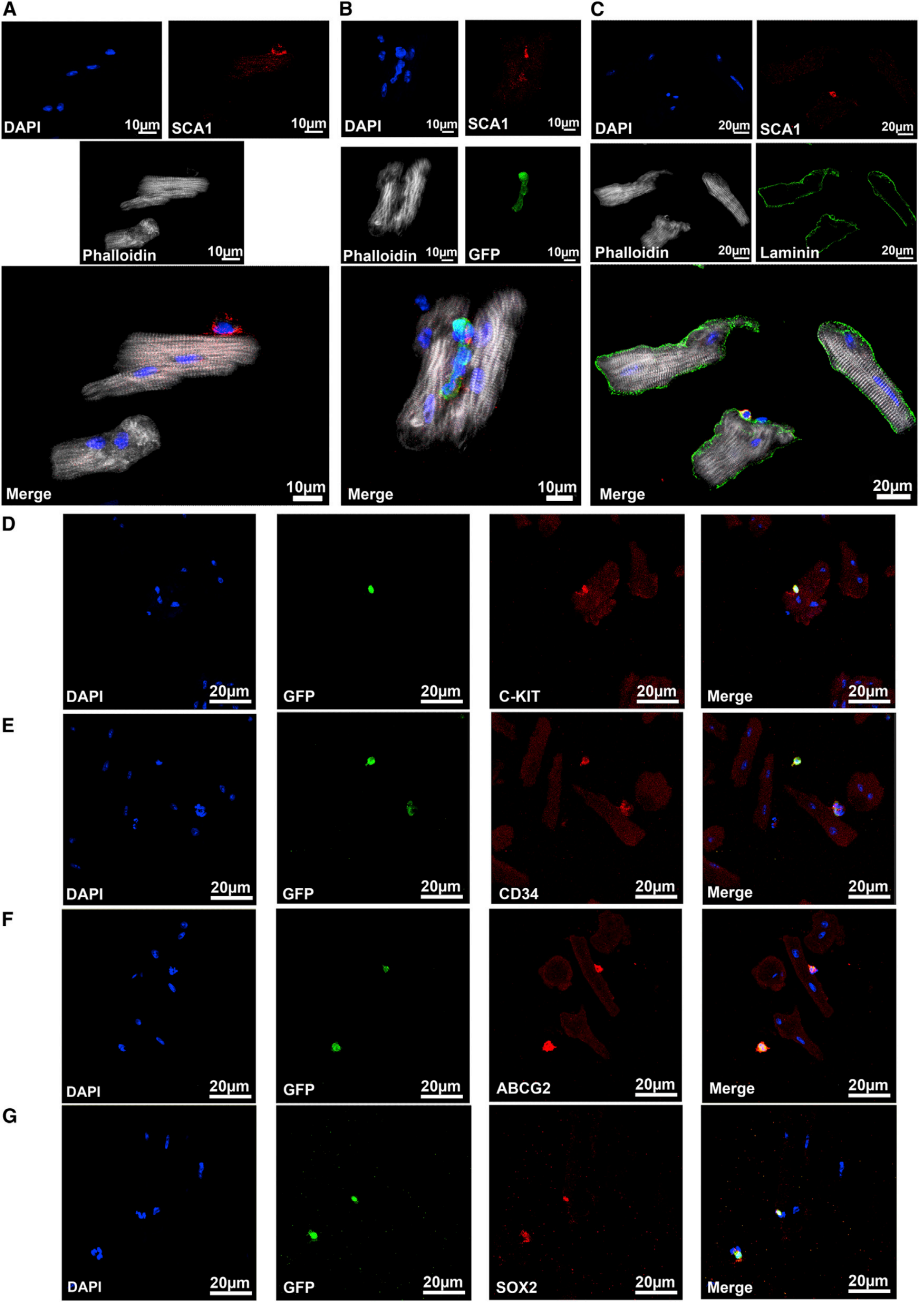
Some *Sca1*-Positive Cells Are Attached to Cardiomyocytes and Situated Underneath the Same Basal Lamina (A) Identification of *Sca1-*positive cells attached to isolated cardiomyocytes with an anti-SCA1 antibody (red). (B) Staining with an anti-SCA1 antibody (red) and with an anti-GFP antibody (green) to simultaneously detect SCA1 and *Sca1*-GFP. (C) Staining with anti-SCA1 antibody (red) and anti-laminin antibody (green) to label *Sca1*-positive cells under the basal lamina. Isolated cells were stained with phalloidin (white) to visualize cardiomyocytes. (D–G) *Sca1-*positive cells attached to cardiomyocytes were identified with an antibody against *Sca1*-GFP (green) and costained with antibodies against C-KIT (red) (D), CD34 (red) (E), ABCG2 (red) (F), or SOX2 (red) (G). All slides were counterstained with DAPI (blue) to visualize nuclei. See also Figure S2.

Numerous reports demonstrated that the heart contains *Sca1*-positive cells (Holmes and Stanford, 2007). To learn more about the identity of cells that express SCA1 in the heart, we stained sections of murine hearts with anti-SCA1 antibody in combination with phalloidin, which provided a general overview of the myocardial morphology. We tested cardiomyocytes at different ages (Figure 1A; Figure S1 available online) and various physiological and pathological conditions (pressure overload induced hypertrophy by transverse aortic constriction [TAC]; Figure 1B) and did not detect any that expressed SCA1 protein; rather, *Sca1-*positive cells were identified in the stromal compartment of the myocardium, probably representing pericytes and mesenchymal and endothelial cells. Although several different SCA1 antibodies and reaction conditions were employed to achieve the highest possible sensitivity, we wondered whether we might have missed some *Sca1*-positive cells. To answer this question, we used *Sca1*-GFP reporter mice, which have been previously demonstrated to faithfully recapitulate endogenous expression of *Sca1* gene (Ma et al., 2002). Cardiomyocytes positive for GFP were not detected both in physiological and pathological conditions (myocardial infarction [MI]; Figures 1C and 1D). Indeed, *Sca1*-GFP positive cells were again seen only in the myocardial stroma, mainly in endothelial cells, as indicated by costaining with BS-1 (*Bandeiraea simplicifolia*) lectin (Figure 1E). To further corroborate these findings and to eliminate possible staining artifacts, cardiomyocytes were isolated from C57BL/6 wild-type and *Sca1*-GFP reporter mice and analyzed for SCA1 expression. No *Sca1*-GFP activity was seen in cardiomyocytes, although we readily detected SCA1 expression in small cells that are closely associated with cardiomyocytes (Figures 2A and 2B).

The association of some *Sca1-*positive cells with cardiomyocytes (identified both with anti-SCA1 antibodies and by *Sca1*-GFP expression) came as a surprise, because stringent conditions were employed to dissociate the myocardium and to obtain single-cell suspension of cardiomyocytes. The location of these *Sca1*-positive cells reminded us of skeletal muscle stem cells called “satellite cells” (Mauro, 1961; Günther et al., 2013). Satellite cells are located on myofibers but below the basal lamina, which contains laminin. Staining for laminin revealed that *Sca1*-positive cells tightly associated with cardiomyocytes are indeed located under the basal lamina (Figure 2C), which might provide a niche for such cells. Quantitative analysis revealed that 430 out of 5,031 isolated cardiomyocytes (8.55%) carried *Sca1*-positive cells under the basal lamina. Interestingly, some *Sca1*-positive cells expressed C-KIT, CD34, ABCG2, and/or SOX2 (Figures 2D–2F), which are typical markers for adult stem cells in various tissues including skeletal muscle (Pannérec et al., 2012; Uchida et al., 2011; Yin et al., 2013). However, the number of SCA1 and C-KIT double positive cells was rather low as determined by flow cytometry (Figure S2). Only 0.4% ± 0.2% of all mononuclear, noncardiomyocytic cells isolated from the myocardium were positive for SCA1 and C-KIT and negative for the endothelial cell marker CD31 and the hematopoietic marker CD45 (CD31^−^/CD45^−^/SCA1^+^/C-KIT^+^). In total, 23.9% ± 3.7% of all mononuclear, noncardiomyocytic cells expressed SCA1, but not CD31 and CD45 (SCA1^+^/CD31^−^/CD45^−^), compared to 0.6% ± 0.3% of C-KIT^+^/CD31^−^/CD45^−^ cells.

### *Sca1*-Derived Cells Give Rise to Cardiomyocytes

**Figure 3:**
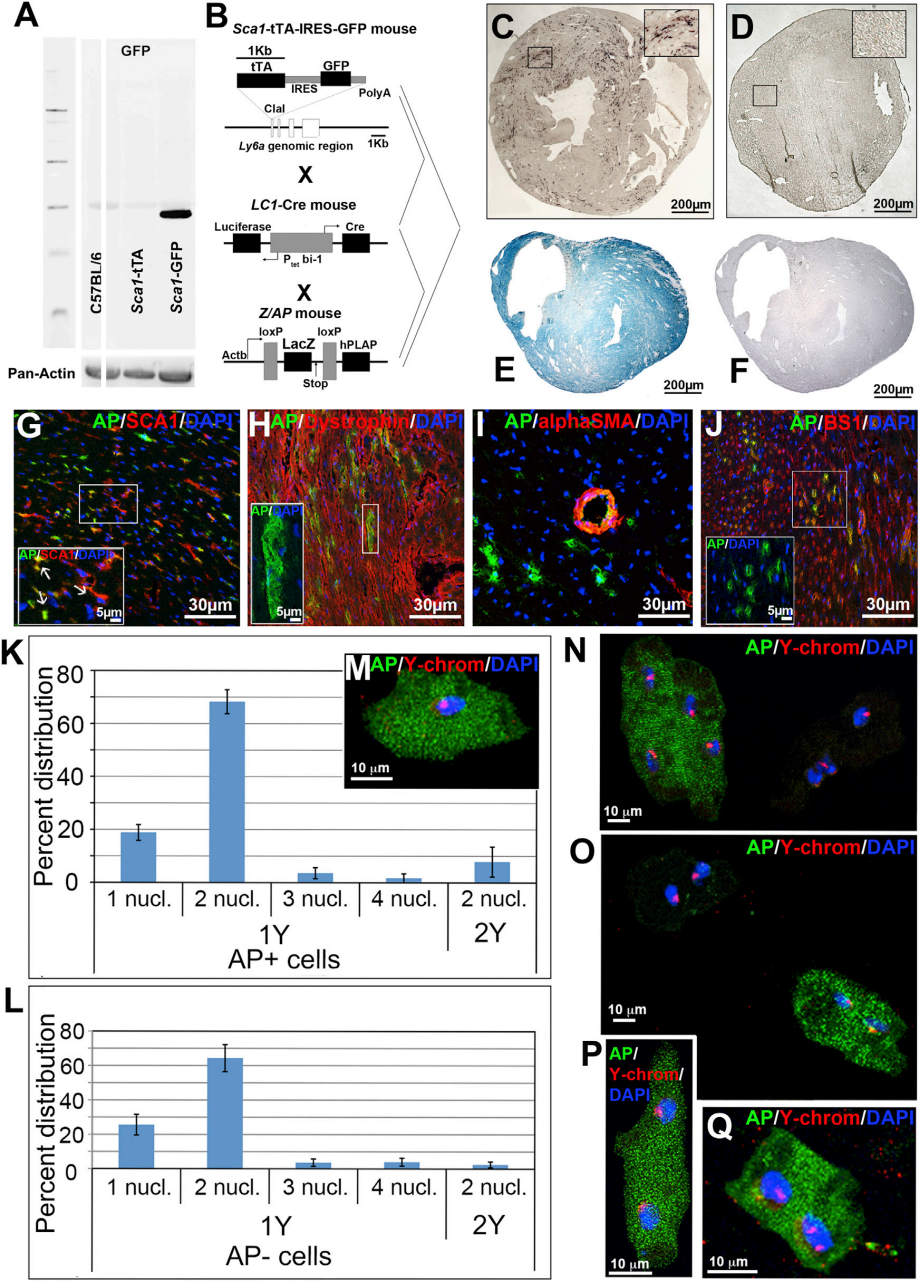
Characterization of *Sca1*-tTA//LC1-Cre//Z/AP Mice (A) Western blot analysis of GFP expression in hearts of C57BL/6 (negative control), *Sca1*-tTA, and *Sca1*-GFP mice (positive control). No GFP expression was monitored in *Sca1*-tTA mice. An antibody against pan-actin was used as a loading control. (B) Scheme for breeding of *Sca1*-tTA//LC1-Cre//Z/AP mice. (C and D) Cross sections of hearts from 2-month-old *Sca1*-tTA //LC1-Cre//Z/AP (C) and LC1-Cre//Z/AP (without *Sca1*-tTA as a negative control) mice (D) stained for AP activity. (E and F) Cross sections of hearts of an 18-month-old female LC1-Cre//Z/AP mouse (without *Sca1*-tTA as a negative control) stained for (E) β-gal (X-Gal) and (F) AP activity (NBT/BCIP). The ubiquitous activity of the lacZ reporter and the lack of AP-reporter gene activity in the absence of *Sca1*-tTA indicate tight, tTA-dependent regulation of the LC1 locus. (G) Triple staining for labeled cells (anti-AP antibody, green), nuclei (DAPI, blue), and SCA1 (anti-SCA1 antibody, red). The arrowhead in the inset indicates a rare SCA1 (marked by a white arrow) and AP (marked by a white arrow) coexpressing cell. (H–J) Triple staining for labeled cells (anti-AP antibody, green), nuclei (DAPI, blue), and the following cell-type-specific markers (red): (H) dystrophin (red) for cardiomyocytes, (I) smooth muscle actin (red) for smooth muscle cells, and (J) BS-1 (red) for endothelial cells. (K and L) Number of nuclei and ploidy of AP-positive (*Sca1*-derived) and AP-negative cardiomyocytes. Adult cardiomyocytes were isolated from male *Sca1*-tTA//LC1-Cre//Z/AP mice and subjected to FISH using a Y chromosome-specific probe followed by immunostaining with an anti-AP antibody and DAPI staining. Mononuclear cardiomyocytes with a single Y chromosome are unlikely to be derived from cellular fusion events. Two *Sca1*-tTA//LC1-Cre//Z/AP male mice were used for this experiment. Error bars represent SDs. (M–Q) Examples of isolated AP-positive and AP-negative cardiomyocytes after FISH with a Y chromosome-specific probe (red). AP expression was detected with an anti-AP antibody (green) and nuclei were visualized by DAPI staining (blue). Note a mononuclear AP-positive cardiomyocyte with a single Y chromosome in (M) and a binuclear cardiomyocyte carrying one nucleus with two Y chromosomes in (Q).

To investigate whether *Sca1*-positive cells in the myocardium contribute to the homeostasis of the myocardium and more specifically to the turnover of cardiomyocytes, we generated a tet-off mouse line (*Sca1*-tTA) employing the same *Sca1* genomic fragment (14 kb of the Ly-6A gene) used to establish the *Sca1*-GFP reporter strain (Ma et al., 2002). Thereafter, *Sca1*-tTA mice were crossed to LC1-Cre mice, in which expression of cre-recombinase is tightly controlled by the tet-system (Schönig et al., 2002), and to Z/AP reporter mice (Lobe et al., 1999). The resulting *Sca1*-tTA//LC1-Cre//Z/AP mice (Figure 3B) allow doxycycline-dependent expression of cre-recombinase (“tet-off”-system), which will permanently activate human placental alkaline phosphatase expression in cells that have expressed *Sca1* (Figure 3C). To rule out potential artifacts, we rigorously analyzed our genetic tracing system. We did not find any alkaline phosphatase (AP)-positive cells in the absence of the *Sca1*-tTA allele (Figure 3D) up to 18 months of age (Figures 3E and 3F), confirming previous results about the regulation of the LC1-Cre locus (Schönig et al., 2002). We did not detect a GFP signal in *Sca1*-tTA mice by immunofluorescence analysis or western blotting, although a GFP gene was inserted behind the tTA gene using an internal ribosome entry site (IRES) (Figure 3A), probably due to the relatively low efficiency of the IRES.

To determine the identity of *Sca1*-derived AP-positive cells in *Sca1*-tTA//LC1-Cre//Z/AP mice, we performed systematic triple staining of consecutive cross sections of the heart using antibodies directed against AP and different cell-type-specific markers (BS-1 for endothelial cells and α-smooth muscle actin for smooth muscle cells). Cardiomyocytes were identified by expression of dystrophin, which delineates cellular boundaries, allowing a clear separation of cardiomyocytes from other cell types (e.g., endothelial cells). In addition, wheat (*Triticum vulgaris*) lectin was used to label all cellular membranes and DAPI was used to visualize nuclei. Initially, we used mice that were not treated with doxycycline and hence produce active tTA, allowing continuous labeling of cells that express *Sca1* gene during the lifetime of the animal, including embryogenesis. We found that virtually all AP-positive cells expressed either BS-1, dystrophin, or α-smooth muscle actin (Figures 3H–3J). Because cardiomyocytes do not express SCA1 under physiological and pathological conditions as described above, we concluded that *Sca1*-derived cells are able to generate cardiomyocytes; we concentrated on this conclusion during the further course of the study.

To rule out that newly emerging AP-positive cardiomyocytes were generated by rare fusion events, we isolated cardiomyocytes from 2-month-old male *Sca1*-tTA//LC1-Cre//Z/AP mice and subjected them to fluorescence in situ hybridization (FISH) analysis using a Y chromosome probe. We found that 18.88% ± 2.97% of isolated AP-positive cardiomyocytes contained one nucleus with one Y chromosome, while 8.06% of all mononucleated cells were AP positive (Figures 3K–3Q). Of note, previous studies have reported conflicting numbers of mononucleated cardiomyocytes, ranging from 2% (Walsh et al., 2010) to 18% (Brodsky et al., 1980), which is most likely due to the different techniques used. Most researchers, who came up with a relatively high number of binucleated cardiomyocytes (>80%) (Brodsky et al., 1980; Liao et al., 2001; Soonpaa et al., 1996; Walsh et al., 2010), employed flow cytometry analysis, whereas our data are based on microscopic analysis of the number of nuclei in isolated cardiomyocytes. Because AP-positive cardiomyocytes with one nucleus and one Y chromosome cannot be generated by a cell fusion event, we concluded that AP-positive cardiomyocytes originated from differentiating *Sca1*-positive cells.

**Figure 4:**
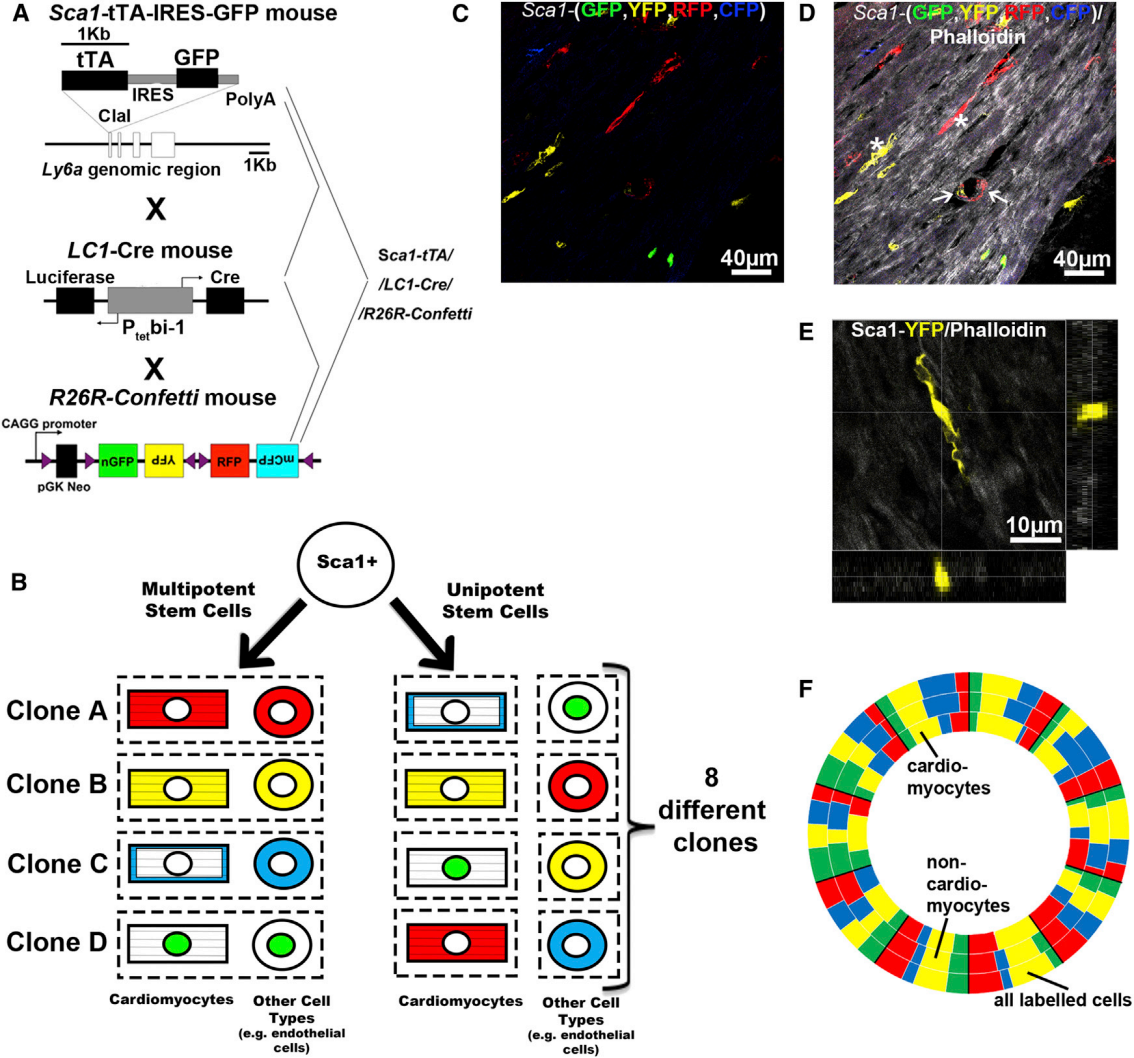
*Sca1*-Derived Cells Undergo Limited Expansion into Distinct Lineages (A) Outline of the strategy to generate *Sca1*-tTA// LC1-Cre//*R26R-Confetti* mice. (B) Schematic representation of potential labeling results using *Sca1*-tTA//LC1-Cre//*R26R-Confetti* mice. Single multipotential *Sca1*-positive clones will give rise to different cell types in the heart (e.g., cardiomyocytes, endothelial cells, and smooth muscle cells) with the same color (e.g., red CSC, red cardiomyocytes, red endothelial cells). Single unipotential *Sca1-*positive clones will give to a single cell type (e.g., cardiomyocytes), all of which have the same color. (C) Representative example of the clonal expansion of labeled *Sca1*-derived cells. (D) Costaining of the same heart section as shown in (C) with phalloidin (in gray). The arrows indicate the contribution of *Sca1*-derived cells to smooth muscle cells and the asterisks to cardiomyocytes. (E) Confocal image of a cardiomyocyte derived from a *Sca1*-derived clone marked in yellow and costained with phalloidin (in gray). (F) Doughnut chart of the distribution of cardiomyocytes (inner doughnut chart), noncardiomyocytes (middle doughnut chart), and all labeled cells (outer doughnut chart) in ten different areas of the myocardium. The size of the labeled boxes represents the frequency of labeled cells in percent. Bold black lines were used to separate each area of counted cells. The unequal distribution of colors for different cell types in most areas excludes that cell types and color are related (p > 0.96 by chi-square test), arguing for distinct progenitor cells for different lineages.

Although previous reports (Oh et al., 2003; Goumans et al., 2007) indicated that *Sca1*-derived cells generate cardiomyocytes and other cell types in vitro, the question remained whether a common *Sca1*-progenitor cell exists in vivo that can give rise to all three lineages of the heart under physiological conditions similar to secondary heart field progenitor cells (Chien et al., 2008). Alternatively, multiple unipotential cell populations all expressing *Sca1* might differentiate into distinct lineages. To explore these possibilities, we took advantage of the *R26R-Confetti* reporter line (Snippert et al., 2010), which, in combination with *Sca1*-tTA and LC1-Cre mice, clonally marks distinctive populations of *Sca1*-derived cells (Figures 4A and 4B). The *R26R-Confetti* allele carries a stochastic multicolor cre-recombinase reporter with four fluorescent proteins (GFP with nuclear localization signal [nGFP], monomeric enhanced yellow fluorescent protein [YFP], red fluorescent protein [RFP], and mCerulean fluorescent protein [CFP]). After stochastic cre-mediated recombination, only one of the four fluorescent proteins will be expressed in each single cell and hence label individual clones (Figure 4C). Costaining with phalloidin and localization of labeled cells clearly identified some *Sca1*-derived cells as cardiomyocytes (Figures 4D and 4E). Interestingly, in the majority of cases, cells from an individual clone belonged to a single lineage. It was rare that cardiomyocytes carrying the same label were found within the same area. Instead, we observed differentially labeled cells of the same lineage in close proximity, which argues for limited expansion of *Sca1*-derived clones and mixing of clones of the same lineage. Furthermore, quantitative evaluation of labeled cells revealed an unequal distribution of the four cellular labels among all marked cells, marked cardiomyocytes, and marked noncardiomyocytes in nine out of ten different areas (Figure 4F). These data indicate that *Sca1*-derived cells possess a strong bias, which prevents cardiomyocyte progenitor cells to generate equal numbers of noncardiomyocytes (such as smooth muscle cells) and vice versa. It should be emphasized that our conclusion about the restricted lineage potential and limited expansion of *Sca1*-derived cells relies on the absence of extensive migration within the myocardium. Since two of the four colors remain in the *R26R-Confetti* allele after initial cre-mediated recombination (blue with red or green with yellow), it is possible that continuous cre activity will induce “flipping” from the active color to the silent color, thereby creating a bias in the distribution of colors (either nGFP/YFP or RFP/mCFP) (Schepers et al., 2012). However, an unequal distribution of cell-type-specific color groups was evident even when only two colors were examined, supporting our initial conclusion about the limited lineage potential of *Sca1*-positive CSCs.

### *Sca1*-Positive Cells Contribute Continuously to Cardiomyogenesis during Physiological Aging

**Figure 5:**
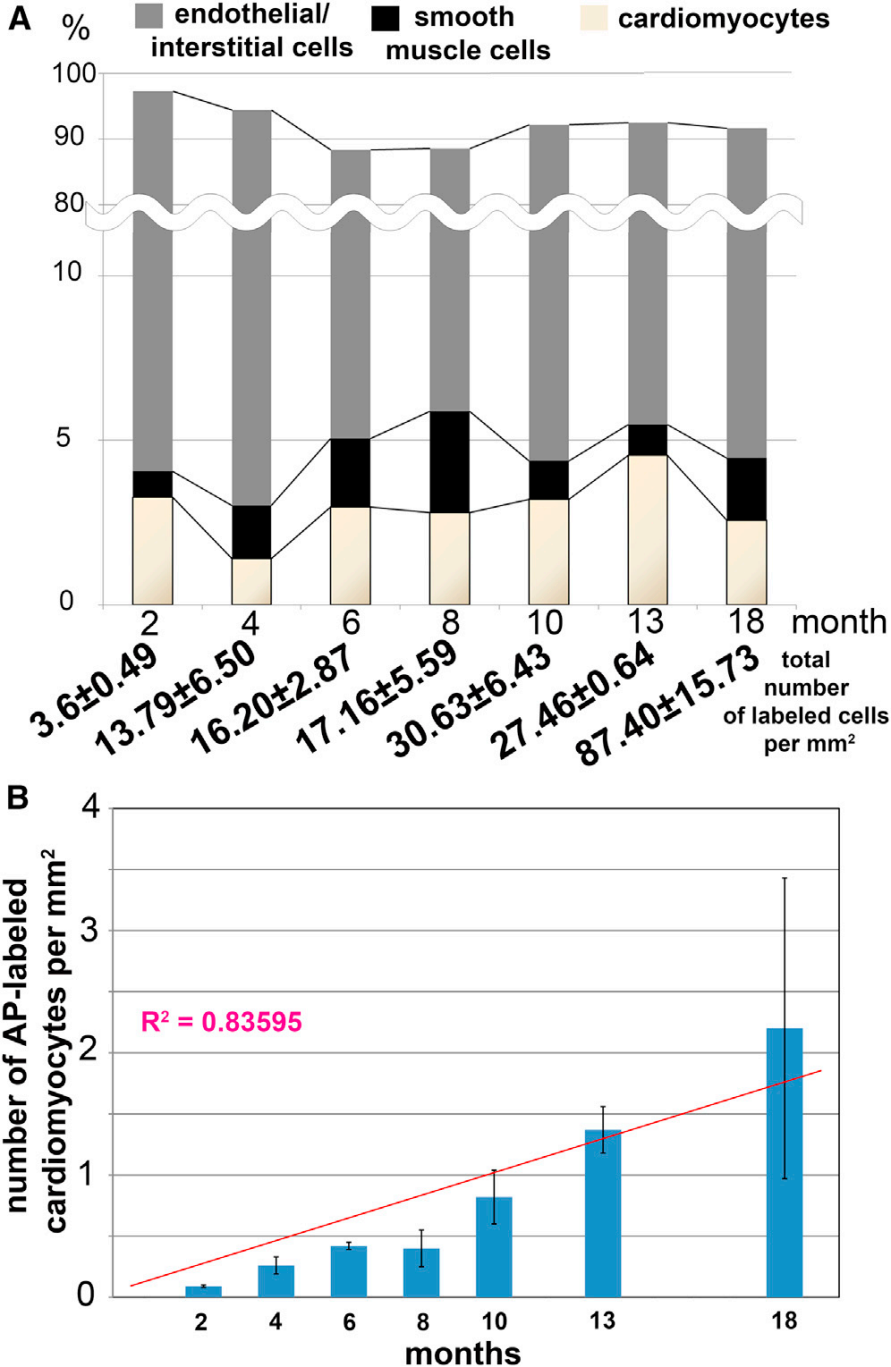
Contribution of *Sca1*-Derived Cells to Different Cell Lineages in the Heart during Normal Aging (A) Relative distribution of AP-labeled endothelial/interstitial cells (gray), smooth muscle cells (black), and cardiomyocytes (yellow) at different postnatal stages in percent. (B) The number of *Sca1*-derived cardiomyocytes increases continuously in the postnatal heart. A nearly linear increase of *Sca1*-derived cardiomyocytes over time was calculated. The number of AP-labeled cardiomyocytes is shown as labeled cells per mm^2^. See also Table S1.

Next, we studied changes in the number of *Sca1*-derived AP-positive cells in the normally aging myocardium, guided by the assumption that homeostatic replacement of cardiomyocytes and other cell types in the heart might result in an increase of AP-positive cells (Figure 5; Table S1). The largest increase was scored in 13- and 18-month-old hearts in which the number of labeled cells increased more than 3-fold (27.46 ± 0.64 and 87.40 ± 15.73 labeled cells per mm^2^ for 13- and 18-month-old hearts, respectively). The relative distribution of different AP-labeled cell types remained similar up to 8 months of the age. From this time point onward, the contribution of cardiomyocytes relative to all labeled cells increased to 4.55% ± 0.87% of cardiomyocytes at 13 months. However, at the age of 18 months, the share of labeled cardiomyocytes dropped to 2.57% ± 1.38%. This might be explained by a major increase of noncardiomyogenic AP-labeled cells between 13- and 18-month-old hearts. Our results suggest that the heart replaces cardiomyocytes at a constant rate during aging from *Sca1*-derived cells until at least 18 months.

### Cardiac Damage and Remodelling Modestly Stimulate the Generation of *Sca1*-Derived Cells

**Figure 6:**
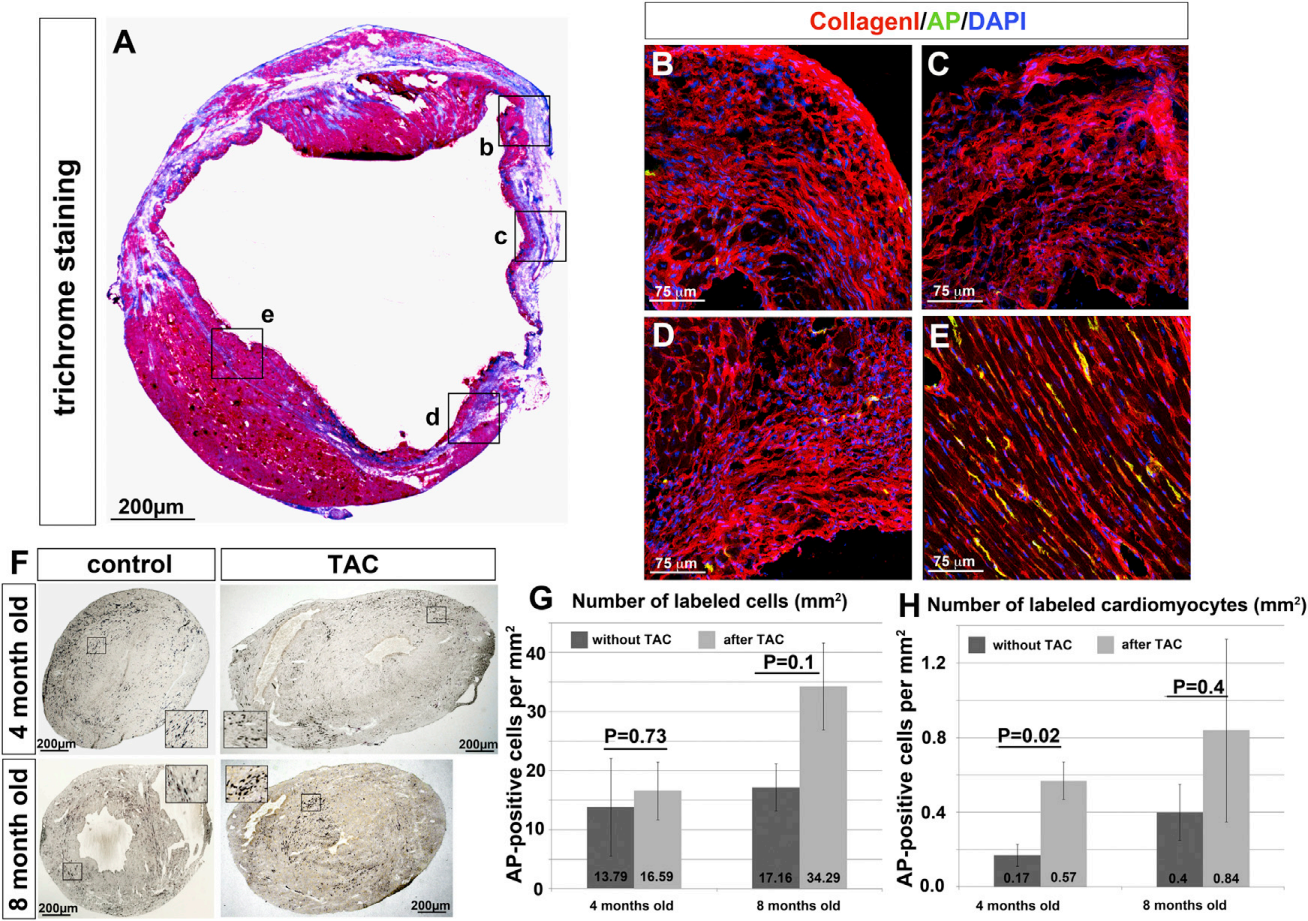
Pressure-Induced Cardiac Hypertrophy but Not Myocardial Infarction Leads to a Moderate Increase of *Sca1*-Derived Cardiomyocytes (A) Trichrome staining of a section from 4-month-old mice (1 month after MI). The areas stained in (B)–(E) are indicated by black boxes. (B–E) Triple labeling of areas from different regions of post-infarcted hearts stained for collagen I (red), AP (anti-AP antibody, green), and nuclei (DAPI, blue). Areas bordering the scar (B and E, identified by massive deposition of collagen I) or within the scar (C) show a reduction of *Sca1*-derived cells. An increase of *Sca1*-derived cells (green) was found in areas showing compensatory hypertrophy (D). (F) AP-staining of cross sections of 4- and 8-month-old mice subjected to TAC at 3 month of age and of control mice. (G) Number of all *Sca1*-derived cells in hearts of TAC-operated and control mice at 4 and 8 months of age. (H) Number of *Sca1*-derived cardiomyocytes in hearts of TAC-operated and of control mice at 4 and 8 months of age (n = 4 mice for 4-month-old TAC-operated samples and n = 3 mice for all the other conditions). Values in (G) and (H) are given in numbers of AP-positive cells per mm^2^. See also Tables S2 and S3.

To investigate the generation of new *Sca1*-derived cardiomyocytes under pathological conditions, we challenged 3-month-old *Sca1*-tTA//LC1-Cre//Z/AP mice by MI. Interestingly, very few AP-positive cells were found in the infarcted area and in the border zone (Figures 6A–6E). In contrast, clusters of AP-positive, BS-1-positive endothelial cells emerged in the septum, whereas the number of AP-positive cardiomyocytes remained constant in the post-MI heart (Figure 6D; Table S2). We concluded that the compensatory hypertrophy of the septum, in response to the loss of myocardial tissue within anterior walls, supports increased recruitment of *Sca1*-derived endothelial cells. In contrast, conditions in the border zone seem to prevent differentiation of *Sca1*-positive cells, which otherwise might have allowed replacement of scar tissue by functional myocardium.

In addition, we asked whether pressure-induced cardiac hypertrophy caused by TAC would promote generation of new *Sca1*-derived cardiomyocytes. Four weeks after the operation, the number of AP-positive cardiomyocytes per mm^2^ had increased more than 3-fold (0.17 ± 0.06 and 0.57 ± 0.10 for the aged-matched and TAC-operated hearts, respectively), while the total number of AP-labeled cells per mm^2^ did not change significantly (18.68 ± 6.40 for TAC-operated and 13.79 ± 6.50 for the aged-matched hearts) (Figures 6F and 6H; Table S3). Five months after the operation, we scored a 2-fold-higher number of AP-positive cardiomyocytes (0.84 ± 0.49 for TAC-operated and 0.4 ± 0.15 for the aged-matched hearts) and of the total number of AP-positive cells (compared to the age-matched hearts [8 months old]) (45.15 ± 7.46 for TAC-operated and 15.23 ± 2.94 for the aged-matched hearts) (Figure 6H). These data indicate that *Sca1*-positive progenitor cells are more responsive to inductive cues that promote differentiation into cardiomyocytes in healthy compared to the diseased myocardium.

### *Sca1*-Derived Cardiomyocytes Are Present in Postnatal Hearts

**Figure 7:**
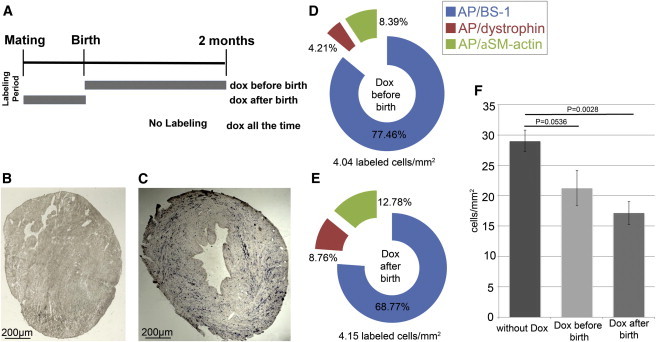
Embryonic and Postnatal *Sca1*-Positive Cells Contribute to Different Cell Lineages in the Heart (A) A schematic timeline of doxycycline (dox) administration in *Sca1*-tTA//LC1-Cre//Z/AP mice. (B) An AP-stained cross section of hearts from mice continuously treated with dox. (C) An AP-stained cross section of hearts from mice treated with dox until birth. (D and E) Relative distributions of AP-labeled endothelial cells, smooth muscle cells, and cardiomyocytes in *Sca1*-tTA-Cre//LC1-Cre//Z/AP mice at 2 month of age that received dox until birth (D) and after birth (E). The statistical test based on Student’s unpaired heteroscedastic t test with a one-tailed distribution indicates that there is no statistically significant difference between dox until birth and after birth for each cell type (cardiomyocytes: p = 0.07; smooth muscle cells: p = 0.148; and endothelial cells: p = 0.179). (F) Administration of dox resulted in decrease of heart sizes. The decrease of heart sizes after dox treatment is reflected by different cross-sectional areas (displayed as mm^2^) of hearts of dox-treated and untreated mice at midventricular levels. See also Table S4.

The continuous increase of *Sca1*-derived cardiomyocytes from 2 to 18 months proved that new cardiomyocytes were generated postnatally (Figure 5) but did not reveal whether these cells were generated directly from *Sca1*-positive progenitor cells present in the postnatal heart (Miles et al., 1997). To address this point, we took advantage of our conditional tet-off system to suppress labeling by administration of doxycycline to pregnant mice either permanently or until shortly after birth (Figure 7A). Staining of hearts from doxycycline-treated animals revealed the absence of AP-positive cells, confirming tight regulation of tTA-dependent cre recombinase expression and the validity of our cell tracing approach (Figure 7B). In a complementary experiment, we suppressed labeling after birth, which restricts tracing to cells that have expressed *Sca1* before birth. At 2 months of age, mice receiving doxycycline either until or after birth were compared (Figures 7C–7E; Table S4). We found that suppression of labeling before birth did not result in a reduction of AP-positive cardiomyocytes but did increase the relative number of cardiomyocytes. Similarly, the suppression of labeling after birth led to an amplification of the number of cardiomyocytes, indicating that some *Sca1*-positive cells were committed to the cardiomyogenic lineage before birth. The increase of cardiomyocytes after doxycycline treatment went along with a general increase of the number of labeled cells per mm^2^. The relative decrease of AP-labeled nonmuscle cells and the increase of cardiomyocytes initially came as a surprise. However, we noted that treatment with doxycycline resulted in a significant reduction in the size of the hearts while the heart/body-weight ratio remained constant (Figure 7F), which might be explained by the suppression of angiogenesis by doxycycline (Fainaru et al., 2008). We concluded that the increase of cardiomyocytes after doxycycline treatment is, at least in part, due to the relative decline of nonmuscle cells and the smaller size of the heart. Taken together, the generation of AP-positive cardiomyocytes after the suppression of labeling before birth and the increase in AP-positive cells after birth clearly demonstrate that a subpopulation of cardiomyocytes is derived from adult *Sca1*-positive cells.

## Discussion

Our study provides clear evidence for the continuous replacement of myocardial cells by *Sca1*-positive CSCs, avoiding the potential problems associated with previously used labeling methods (Bergmann et al., 2009; Kajstura et al., 2010). Yet it is clear that our strategy has limitations. We focused on the contribution of a distinct subset of potential CSCs, which automatically excludes other putative CSCs. Therefore, we might have systematically underestimated the extent of myocardial turnover. Moreover, there is no consensus about the rate of cardiac regeneration in mammals. Some studies demonstrated very little, if any, turnover of murine cardiomyocytes during normal aging, whereas other studies in humans reported replacement of about 45% of all cardiomyocytes during the whole lifetime or even a full replacement of all cardiomyocytes within 5 years (reviewed in Laflamme and Murry, 2011). All these issues make it difficult to determine the relative contribution of *Sca1*-derived cells to myocardial renewal. Under any circumstance, our results demonstrate that at least a few percent of cardiomyocytes are replaced by *Sca1*-derived cardiomyocytes during normal aging.

Our analysis revealed that *Sca1*-positive cells in the heart can give rise to cardiomyocytes. It also seems likely that *Sca1*-positive stromal cells differentiate to endothelial cells (Hoch et al., 2011), but unequivocal proof for this hypothesis is difficult to obtain because some endothelial cells also express *Sca1*. Furthermore, the use of the *R26R-Confetti* reporter line enabled us to demonstrate that individual *Sca1*-positive cells own a restricted potential for expansion and usually give rise only to a single cell type. This feature distinguishes adult CSCs from embryonic secondary heart field progenitor cells, which have the potential to differentiate into cardiomyocytes, smooth muscle, or endothelial cells (Chien et al., 2008). Despite the presence of abundant numbers of *Sca1*-positive cells in the heart, only a small subset of *Sca1*-positive cells differentiated into cardiomyocytes in our study. This observation might explain the limited contribution of *Sca1*-positive CSC to the adult myocardium. Furthermore, it is tempting to speculate that heterogeneity of *Sca1*-positive cells is one of the major reasons why different groups obtained conflicting data regarding the differentiation potential of *Sca*1-positive cells (Hoch et al., 2011; Holmes and Stanford, 2007; Matsuura et al., 2004; Tateishi et al., 2007). Clearly, further efforts are needed to characterize different populations of CSCs and to explore how endogenous CSCs can be protected and stimulated in order to enhance cardiac repair beyond normal limits.

## Experimental Procedures

### Mice

Triple-transgenic mice were obtained by breeding *Sca1*-tTA with LC1-Cre (Schönig et al., 2002) and Z/AP (Lobe et al., 1999) or *R26R-Confetti* (Snippert et al., 2010) reporter mice. *Sca1*-tTA mice were generated by pronucleus injection using standard procedures. Construction of the transgene was based on the insertion of the tetracycline transactivator (tTA)-IRES-GFP-polyA cassette (∼2.6 Kb) into the ClaI site of pPOLYIII-*Ly6A*, which contains sequences of the *Sca1* genomic region (Ma et al., 2002). All strains were maintained on a C57BL/6 genetic background after backcrossing. Doxycycline was administered in drinking water at 1 mg/ml together with 30 mg/ml sucrose.

### Myocardial Infarction and Transverse Aortic Constriction

MI was achieved by permanent ligation of the left anterior descending coronary artery as described previously (Belema-Bedada et al., 2008). Transverse aortic constriction was accomplished by applying a Weck hemoclip to the proximal aorta, resulting in an acute left-ventricular pressure overload (Kreymborg et al., 2010). All animal experiments in this study were performed with approval of the local animal care committee.

### Antibody, Histochemical, and Histological Staining

Dissected hearts were washed in PBS, snap-frozen in liquid nitrogen, and stored at −80°C until further use. 6 μm sections were prepared on a cryostat before fixation in 4% paraformaldehyde. Reaction of sections with primary and secondary antibodies followed established protocols (Belema-Bedada et al., 2008). Nuclei were stained with DAPI. All antibodies used in this study are described in Table S5.

To stain for AP activity, cryosections were fixed with 0.4% glutaraldehyde, heated at 70°C for 30 min in PBS, and incubated in NTMT buffer for 30 min at room temperature. The color was developed in NBT/BCIP staining solution at 37°C for 2 hr.

Double staining for X-Gal and SCA1 protein was achieved by performing the X-Gal reaction first. After washing in PBS three times, sections were permeabilized with 0.05% Triton-X at room temperature for 10 min and washed in PBS three times. The antibody against SCA1 (Abcam) was diluted at 1:100 in 0.005% Triton-X and 0.1% BSA and incubated at 4°C for overnight. The next day, sections were washed three times in PBS. Bound antibody was visualized by the rat Vectastain Elite ABC kit (Vector Laboratories, #PK-6104) using the DAB Plus Substrate Staining System (Thermo Scientific, #TA-060-HDX). The reaction with DAB substrate was performed for 5 min and stopped by washing slides with tap water.

### Counting of Immunofluorescence Stained Cells

To determine the numbers of differentially stained cell types, at least 20 sections were prepared for each staining from each individual heart by collecting ten consecutive sections on individual slides. Next, the procedure was repeated, yielding ten slides with two sections on each slide. Slides were reacted with a series of antibodies indicated in the main text. In a stained section, all labeled cells were counted by switching between fluorescent filters (blue for DAPI, green for AP, and red for cell type) to make sure that individual cells were only counted once. In some cases, when cells were lying on top of each other (especially in the case of endothelial cells), the stage of fluorescent microscope was adjusted, guided by the nuclear DAPI stain, to make sure that individual cells were counted. To avoid investigator-dependent bias, all sections were independently counted by two different persons and checked for consistency. When numbers of counted cells differed significantly between two sections on one slide, the same staining scheme was repeated with an extra slide. To calculate the percentage of cell types, the number of AP-positive, cell-type-marker-positive cells was divided by number of AP-positive, lectin-positive cells. To derive p values, the Wilcoxon Mann-Whitney test was applied in R. Results are presented as mean ± SEM.

To define a clonal expansion of labeled Sca1-derived cells in the hearts of Sca1-tTA//LC1-Cre//R26R-Confetti mice, ten fields were randomly chosen from each section of the heart using ×40 magnification on confocal microscope and a picture was taken. This picture is defined as one area. Then, the number of labeled cells with one of four colors was counted separately. To identify labeled cardiomyocytes, merged images were examined for phalloidin-positive cells.

### Confocal Microscope

Pictures were acquired using a confocal microscope (Leica SP2). Each picture was been taken using scan mode xyz, format of 1,024 × 1,024 pixels, speed at 400 Hz, image x and y dimensions of 238.10 μm, voxel-size of 231.51 × 231.51 nm × nm, average line 1, average frame 3, and pinhole 1. The z stack was done with a pitch of 0.5 μm (the stack calculation was of 10 μm), and 20 pictures were acquired. Snapshots of the z stack pictures were done using Leica Confocal software. The objectives used were HCXPL APO CS 40× 1.25 oil and HCXPL APO lbd BL 63× 1.4 oil. The lasers considered were 405 nm, 488 nm, 514 nm, 564 nm, 564 nm, and 633 nm. Adobe Photoshop CS4 was used to adjust the brightness and contrast of the picture (in RGB color mode), the white and black levels, and the merge of the different channels, when necessary. Each picture was adjusted to a width of 6 cm and a resolution of 300 pixels/inch.

### Cardiomyocyte Isolation

Cardiomyocytes were isolated as previously published (O’Connell et al., 2003). Isolated cardiomyocytes were allowed to attach to chamber slides coated with laminin overnight followed by staining of attached cardiomyocytes.

### Fluorescent In Situ Hybridization

Isolated cardiomyocytes were stained with mouse IDetect Chr Y FISH Paint Probe Red (Labs Inc Biotechnology) as described previously (Zheng et al., 2007). Immunofluorescence staining of FISH-hybridized cells was performed as described above. For counting, ten randomly selected fields were examined.

### Western Blot Analysis

Tissue samples were homogenized by sonication in the presence of proteinase inhibitors and heated for 1 min at 99°C. Protein samples (20 μg) were resolved on a 4%–12% SDS polyacrylamide gradient gel (Life Technologies) and blotted onto a nitrocellulose membrane. Bound antibodies were visualized using VersaDoc MP Systems (Bio-Rad) and the Femto detection kit (Thermo Scientific) according to the manufacturer’s instructions.

#### Flow Cytometry Analysis

Adult mouse hearts (female, 8 weeks of age) were cut into small pieces (1–2 mm in diameter) and digested with collagenaseD (Roche) and DNase I (Serva) for 30 min at 37°C. Red blood lysis was performed using sterile filtered deionized water followed by two washing steps with PBS. Debris was removed with a 30 μm filter. The resulting single-cell suspension was preincubated with blocking rat-mouse antibodies to the FcRγ III/II receptor (clone 2.4G2; BD Biosciences) before staining with primary antibodies. The following conjugated primary antibodies were used: fluorescein isothiocyanate mouse anti-CD45.2 (clone 104), phycoerythrin-conjugated anti-C-KIT (2B8), peridinin chlorophyll protein-eFluor710 conjugated anti-CD31 (clone 390), and allophycocyanin-conjugated anti-Sca1 (Ly-6A/E, clone D7; all from eBioscience). FACS data were obtained by analyzing 100,000 live cells (DAPI negative) in the stem cell gate (FSC-A and SSC-A) using appropriate isotype controls. Measurements were accomplished with a FACS Canto II instrument (BD Biosciences) and analyzed with FlowJo software (TreeStar).
