# Supplementary material for: Sca1-Derived Cells Are a Source of Myocardial Renewal in the Murine Adult Heart
Source: Stem Cell Reports. 2013 Oct 24;1(5):397–410. doi: 10.1016/j.stemcr.2013.09.004 (PMC3841250; doi:10.1016/j.stemcr.2013.09.004)
Supplement: Document S1. Figures S1 and S2 and Tables S1–S5 [file mmc1.pdf]

Stem Cell Reports, Volume *1*

Supplemental Information

Sca1-Derived Cells Are a Source of Myocardial Renewal in the Murine Adult Heart

Shizuka Uchida, Piera De Gaspari, Sawa Kostin, Katharina Jenniches, Ayse Kilic, Yasuhiro Izumiya, Ichiro Shiojima, Karsten grosse Kreymborg, Harald Renz, Kenneth Walsh, and Thomas Braun

INVENTORY OF SUPPLEMENTAL INFORMATION

- **Figure S1, related to Figure 1. Expressions of SCA1 protein in hearts.** This supplementary figure shows staining of anti-SCA1 antibody of the hearts of different ages to supplement the information provided in Figure 1.
- **Figure S2, related to Figure 2. Numbers of stem-cell-marker-positive cells in the heart.** This supplementary figure includes flow cytometry analysis of SCA1-positive cells in conjugation to another stem cell marker C-KIT to supplement the information regarding the small Sca1-expressing cells attached to cardiomyocytes.
- **Table S1, related to Figure 5. Numbers of Sca1-derived (AP-positive) endothelial cells (BS-1-positive, indicated in the "AP/BS-1" column), cardiomyocytes, (dystrophin-positive, indicated in the "AP/Dystrophin" column), and smooth muscle cells (alpha-smooth-muscle-actin- (αSM)-positive, indicated in the "AP/ α-SM" column) at different ages per mm^2.** This supplementary table displays the summary of counting of cell-type-specific labeled cells per mm^2 as well as in percentage for cardiomyocytes and results of statistical test.
- **Table S2, related to Figure 6. Numbers of Sca1-derived (AP-positive) endothelial cells (BS-1 positive), cardiomyocytes, (dystrophin-positive), and smooth muscle cells (α-SM-actin positive) at 4 months of ages without and myocardial infarction per mm^2.** This supplementary table shows the number of labeled cells normalized to mm^2 of each cell type for each individual mouse used for this study (Figure 6D).

- **Table S3, related to Figure 6. Numbers of Sca1-derived (AP-positive) endothelial cells (BS-1 positive), cardiomyocytes, (dystrophin-positive), and smooth muscle cells (α-SM-actin positive) at different ages without and with transverse aortic constrictions (TAC) per mm^2.** The numbers shown in this supplementary table are the raw data used to generate Figure 6G & H.
- **Table S4, related to Figure 7. Numbers of *Sca1*-derived (AP-positive) endothelial cells (BS-1 positive), cardiomyocytes, (dystrophin-positive), and smooth muscle cells (α-SM-actin positive) *Sca1*-tTA-Cre//LC1-Cre//Z/AP mice at 2 month of age that received doxycycline (Dox) until birth or after birth as well as age-matched control per mm^2.** The numbers shown in this supplementary table are the raw data used to generate Figure 7D&E.
- **Table S5. List of antibodies used in this study.**

SUPPLEMENTARY INFORMATION

SUPPLEMENTARY FIGURES

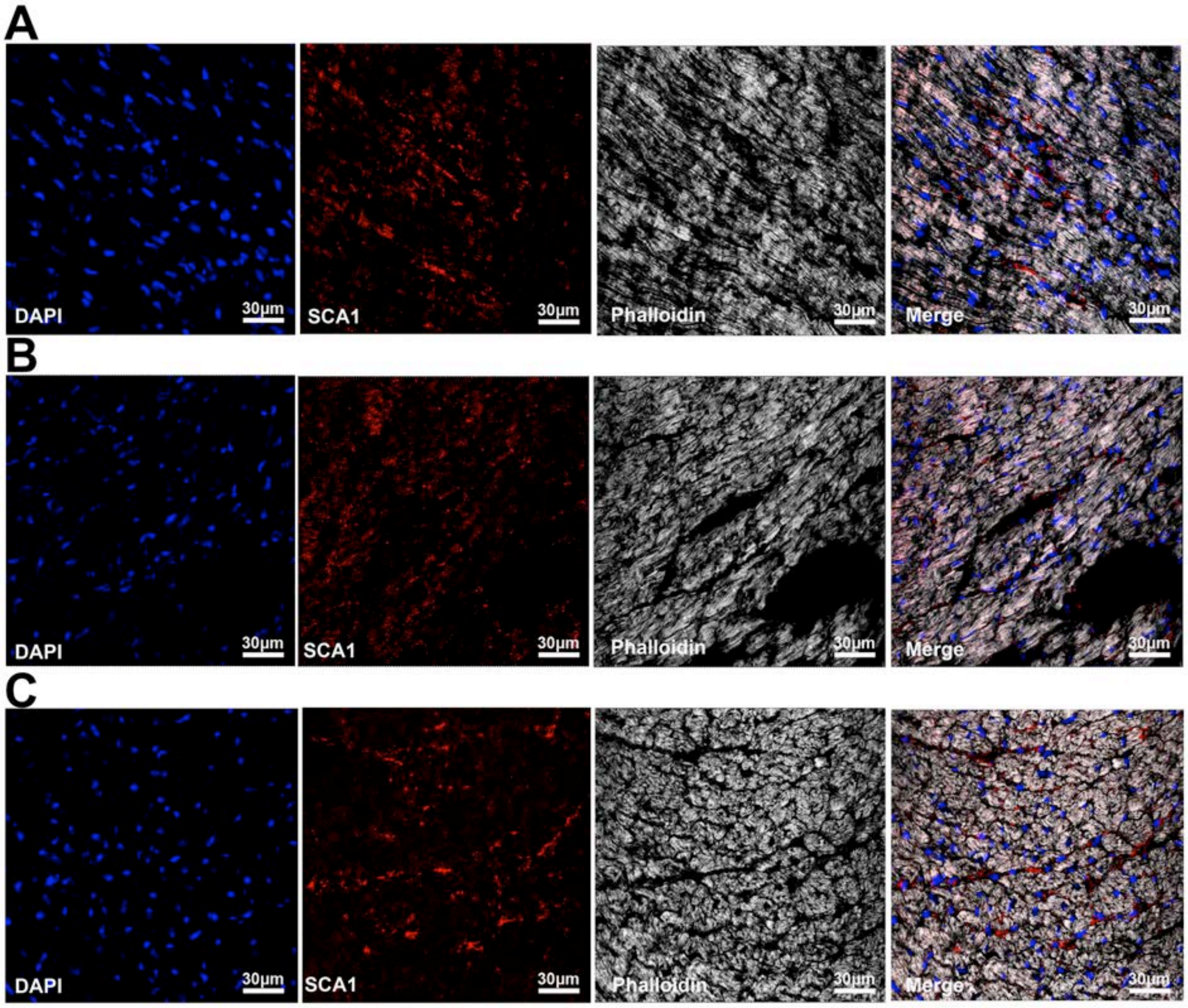

Figure S1, related to Figure 1. Expressions of SCA1 protein in hearts. (A-C) Tissue sections were stained with anti-SCA1 antibody (red) and phalloidin (white) to visualize cardiomocytes from murine hearts of various ages as follows: (A) 2-; (B) 10-; and (C) 18-month old normally aged mice. All the above sections were counterstained with DAPI to visualize nuclei (blue).

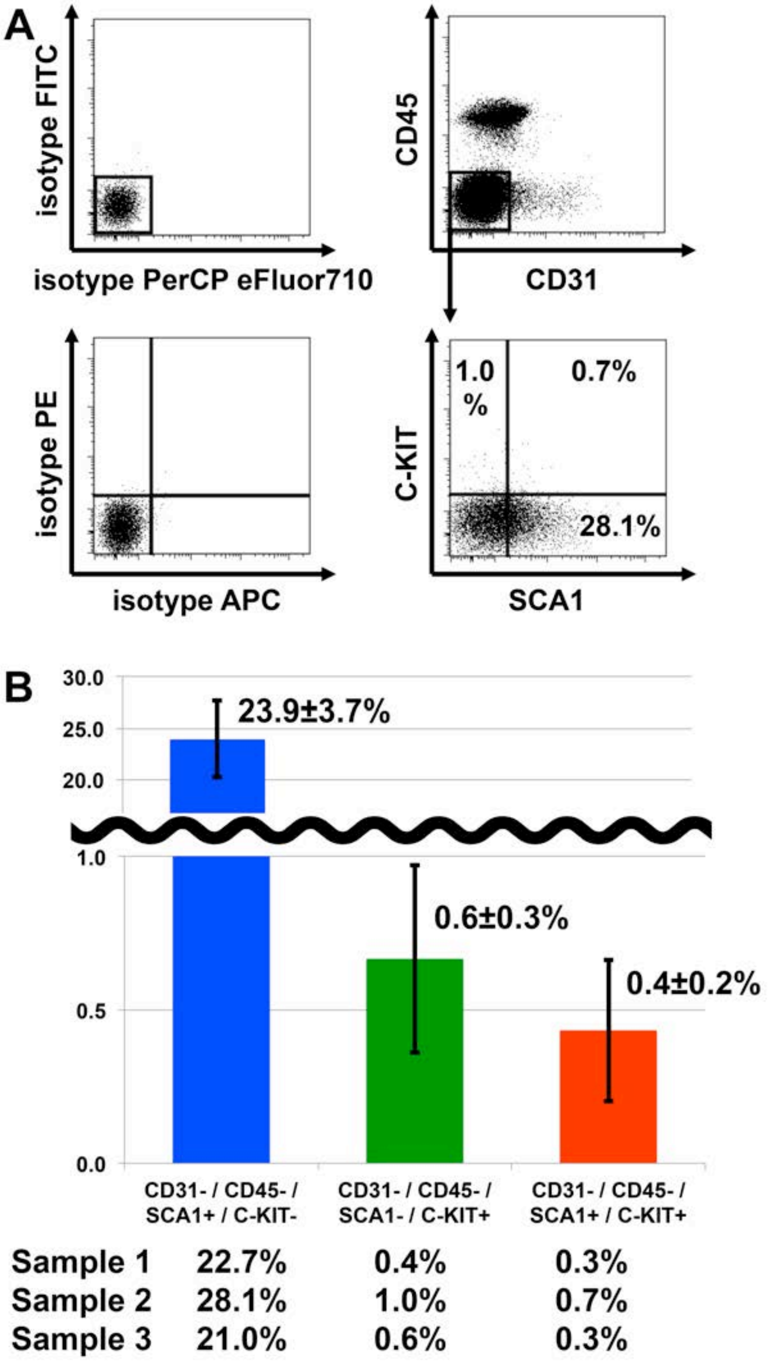

Figure S2, related to Figure 2. Numbers of stem-cell-marker-positive cells in the heart. (A) Representative flow cytometry analysis of $SCA1^+$ and $C\text{-}KIT^+$ progenitor cells. (B) Bar graph showing the average values of each population of cells (n=3 independent preparations). The following conjugated primary antibodies were used: fluorescein isothiocyanate mouse anti-CD45.2 (clone 104), phycoerythrin-conjugated anti-C-KIT (2B8), peridinin chlorophyll protein-eFluor710 conjugated anti-CD31 (clone 390) and allophycocyanin-conjugated anti-SCA1 (Ly-6A/E, clone D7; all from eBioscience). Standard deviations are shown with "±".

TABLES

	AP/ BS-1 (cells/ mm^2)	AP/ Dystrophin (cells/ mm^2)	AP/ α -SM-actin (cells/ mm^2)	Total Labeled Cells (cells/ mm^2)	t-test	ANOVA	n
**2 m.o.**	4.13±0.76	0.09±0.01	0.05±0.05	3.60±0.49	---	0.2546	3
**4 m.o.**	9.65±3.63	0.17±0.06	0.17±0.03	13.79±6.50	0.164	0.3074	3
**6 m.o.**	18.32±6.00	0.42±0.03	0.35±0.12	16.20±5.42	0.0198	0.6093	2
**8 m.o.**	15.23±2.94	0.40±0.15	0.47±0.18	17.16±5.59	0.4544	0.5013	3
**10 m.o.**	33.50±10.20	0.82±0.22	0.33±0.12	30.63±6.43	0.0958	1	3
**13 m.o.**	36.22±3.67	1.37±0.19	0.24±0.10	27.46±0.64	0.0655	0.4714	3
**18 m.o.**	67.63±11.88	2.20±1.23	1.50±0.16	87.40±15.73	0.275	0.0132	4

Table S1, related to Figure 5. Numbers of Sca1-derived (AP-positive) endothelial cells (BS-1-positive, indicated in the "AP/BS-1" column), cardiomyocytes, (dystrophin-positive, indicated in the "AP/Dystrophin" column), and smooth muscle cells (alpha-smooth-muscle-actin- (α -SM-actin)-positive, indicated in the "AP/ α -SM" column) at different ages per mm^2. In the "Total Labeled Cells", numbers of AP-positive, lectin-positive cells are shown. All labeled cells in the respective sections were counted. SEM values are indicated. The percentages of cardiomyocytes in this table were calculated after determining the number of labeled cells per mm^2. In the "t-test" column, Student's unpaired heteroscedastic t-Test with a one-tailed distribution was performed comparing two age groups (e.g. 2 m.o. compared to 4 m.o.; 4 m.o. compared to 6 m.o.). In the "ANOVA" column, p-values derived from one-way analysis of variance (ANOVA) for independent samples (one age group compared to all the other age groups combined) using the numbers of AP-labeled cardiomyocytes are shown. In the "n" column, the number of mice used for each category is shown.

	AP/BS-1	AP/dystrophin	AP/ α -SM-actin	Total number
4 months control	3.29	0.08	0.12	4.98
	15.86	0.28	0.23	26.47
	9.81	0.15	0.17	9.91
	9.65±3.63	0.17±0.06	0.17±0.03	13.79±6.50
4 months Infarction	9.09	0.15	0.3	8.4
	2.29	0.14	0.15	3.41
	10.55	0.19	0.26	5.7
	46.35	0.4	0.57	5.36
	31.98	0.33	0.59	39.46
	18.58	0.26	0.96	17.93
	19.80±6.74	0.24±0.04	0.47±0.12	1782±6.09

Table S2, related to Figure 6. Numbers of *Sca1*-derived (AP-positive) endothelial cells (BS-1 positive), cardiomyocytes (dystrophin-positive), and smooth muscle cells (α -SM-actin positive) at 4 months of ages without and myocardial infarction per mm^2. Ligation of the LAD was performed at 3 months of age. Data from individual animals and average values (in gray boxes) are shown. SEM values and sample numbers are indicated. All labeled cells in the respective sections were counted.

	AP/BS-1	AP/dystrophin	AP/α-SM-actin	Total number
4 months control	3.29	0.08	0.12	4.98
	15.86	0.28	0.23	26.47
	9.81	0.15	0.17	9.91
	9.65±3.63	0.17±0.06	0.17±0.03	13.79±6.50
4 months TAC	12.08	0.55	0.05	15.18
	27.87	0.82	0.09	16.58
	30.82	0.6	0.82	29.24
	3.98	0.33	0.26	5.36
	18.68±6.40	0.57±0.10	0.3±0.18	16.59±3.87
8 months control	18.77	0.7	0.13	23.47
	9.39	0.26	0.53	9.76
	17.54	0.26	0.76	18.27
	15.23±2.94	0.40±0.15	0.47±0.18	17.16±5.59
8 month TAC	36.01	1.81	0.92	25.9
	59.93	0.47	2.76	48.96
	39.5	0.24	1.37	28.0
	45.15±7.46	0.84±0.49	1.68±0.42	34.29±12.59

Table S3, related to Figure 6. Numbers of *Sca1*-derived (AP-positive) endothelial cells (BS-1 positive), cardiomyocytes, (dystrophin-positive), and smooth muscle cells (α-SM-actin positive) at different ages without and with transverse aortic constrictions (TAC) per mm^2. TAC operation was performed at 3 months of age. Data from individual animals and average values (in gray boxes) are shown. SEM values and sample numbers are indicated. All labeled cells in the respective sections were counted.

	AP/BS-1	AP/dystrophin	AP/α-SM-actin	Total number
2 months control	3.85	0.07	0	4.33
	5.57	0.10	0.14	4.66
	2.96	0.11	0	1.82
	4.13±0.76	0.09±0.01	0.05±0.05	3.60±0.49
2 months Dox Before Birth	3.99	0.16	0.41	3.49
	3.07	0.13	0.50	4.08
	2.33	0.12	0.16	2.65
	5.46	0.33	0.28	5.86
	3.50	0.17	0.53	4.14
	3.67±0.52	0.18±0.04	0.38±0.07	4.04±0.53
2 months Dox After Birth	0.93	0.11	0.17	1.22
	11.26	0.23	0.23	13.01
	0.47	0.23	0.28	0.99
	4.52	0.24	0.49	5.65
	1.89	0.22	0.18	2.02
	1.33	0.133	0.23	2.03
	3.40±1.68	0.19±0.02	0.26±0.05	4.15±1.90

Table S4, related to Figure 7. Numbers of *Sca1*-derived (AP-positive) endothelial cells (BS-1 positive), cardiomyocytes, (dystrophin-positive), and smooth muscle cells (α-SM-actin positive) *Sca1*-tTA-Cre//LC1-Cre//Z/AP mice at 2 month of age that received doxycycline (Dox) until birth or after birth as well as age-matched control per mm^2. Data from individual animals and average values (in gray boxes) are shown. SEM values and sample numbers are indicated. All labeled cells in the respective sections were counted.

**Target**	**Company**	**Catalog**	**Application**	**Dilution**
Alkaline phophatase, placental (PLAP)	Accurate Chemical & Scientific Corp.	YSRTAHP537	Immunohistochemistry	1:50
Ly-6A/E	BD Pharmingen	553333	Immunohistochemistry	1:100
Ly-6A/E	Abcam	ab51317	Immunohistochemistry	1:100
Collagen Type I Biotin Conjugate	Rockland	600-406-103	Immunohistochemistry	1:100
Lectin from Bandeiraea simplicifolia TRITC Conjugate (BS-1)	Sigma-Aldrich	L5262	Immunohistochemistry	1:20
dystrophin (clone: MandyS8)	Sigma-Aldrich	D8168	Immunohistochemistry	1:50
α -smooth muscle actin (Cy3-conjugated)	Sigma-Aldrich	C6198	Immunohistochemistry	1:300
Lectin from Triticum vulgaris TRITC Conjugate (Lectin)	Sigma-Aldrich	L5266	Immunohistochemistry	1:20
Phalloidin (labeled with Alexa Fluor 633)	Life Technologies		Immunohistochemistry	1:300
GFP	Abcam	ab6556	Western blotting	1:10,000
Pan-Actin	Cell Signaling Technology	4968	Western blotting	1:1,000
fluorescein isothiocyanate mouse CD45.2 (clone 104)	eBioscience	11-0454	Flow Cytometry	1:100
peridinin chlorophyll protein-eFluor710 conjugated anti-CD31 (clone 390)	eBioscience	46-0311	Flow Cytometry	1:100
allophycocyanin-conjugated anti-SCA1 (Ly-6A/E, clone D7)	eBioscience	17-5981	Flow Cytometry	1:100
Anti-Mouse CD117 (C-KIT) PE	eBioscience	12-117181	Flow Cytometry Immunohistochemistry	1:100

Rat IgG2a K Isotype Control PerCP-eFluor® 710	eBioscience	46-4321-82	Flow Cytometry	1:100
FITC Anti-Mouse IgG2a (clone: R19-15)	BD	1031751	Flow Cytometry	1:100
PE Rat IgG2b, k Isotype Control (clone: A95-1)	BD	553989	Flow Cytometry	1:100
Rat IgG2a K Isotype Control APC	eBioscience	17-4321	Flow Cytometry	1:100
ABCG2	Abcam	ab24115	Immunohistochemistry	1:100
GFP	Invitrogen	A-21311	Immunohistochemistry	1:100
CD34	Abcam	ab81289	Immunohistochemistry	1:100
SOX2	Abcam	ab97959	Immunohistochemistry	1:100
Anti-Mouse Ly-6A/E PE	eBioscience	12-5981-82	Immunohistochemistry	1:100

Table S5. List of antibodies used in this study.
